# An Epigenetic *LINE-1*-Based Mechanism in Cancer

**DOI:** 10.3390/ijms232314610

**Published:** 2022-11-23

**Authors:** Patrizia Lavia, Ilaria Sciamanna, Corrado Spadafora

**Affiliations:** 1 Institute of Molecular Biology and Pathology (IBPM), CNR Consiglio Nazionale delle Ricerche, c/o Department of Biology and Biotechnology, Sapienza University of Rome, 00185 Rome, Italy; 2 Center for Animal Research and Welfare (BENA), ISS Istituto Superiore di Sanità, 00161 Rome, Italy; 3 Institute of Translational Pharmacology (IFT), CNR Consiglio Nazionale delle Ricerche, 00133 Rome, Italy

**Keywords:** cancer genesis and progression, *LINE-1* retrotransposons, reverse transcriptase (RT), RT inhibitors, autophagy, nuclear lamina, chromatin, genome expression, embryogenesis

## Abstract

In the last fifty years, large efforts have been deployed in basic research, clinical oncology, and clinical trials, yielding an enormous amount of information regarding the molecular mechanisms of cancer and the design of effective therapies. The knowledge that has accumulated underpins the complexity, multifactoriality, and heterogeneity of cancer, disclosing novel landscapes in cancer biology with a key role of genome plasticity. Here, we propose that cancer onset and progression are determined by a stress-responsive epigenetic mechanism, resulting from the convergence of upregulation of *LINE-1* (*long interspersed nuclear element 1*), the largest family of human retrotransposons, genome damage, nuclear lamina fragmentation, chromatin remodeling, genome reprogramming, and autophagy activation. The upregulated expression of *LINE-1* retrotransposons and their protein products plays a key role in these processes, yielding an increased plasticity of the nuclear architecture with the ensuing reprogramming of global gene expression, including the reactivation of embryonic transcription profiles. Cancer phenotypes would thus emerge as a consequence of the unscheduled reactivation of embryonic gene expression patterns in an inappropriate context, triggering de-differentiation and aberrant proliferation in differentiated cells. Depending on the intensity of the stressing stimuli and the level of *LINE-1* response, diverse degrees of malignity would be generated.

## 1. Introduction: The “War on Cancer” and Its Legacy

Over half a century ago, the “war on cancer” was declared in the US (National Cancer Act, 1971). That event, despite being at the time strongly biased by the assumption that cancer was generated only or prevalently by tumorigenic viruses, started an era of striking development in molecular cancer research and ushered unprecedented economic and scientific efforts recognizing that the only way to defeat cancer was to better understand it. The war on cancer has traditionally focused on chemotherapy and radiotherapy and, more recently, on targeted therapy with the aim of developing drugs that block cancer hallmarks [1,2,3,4], based on the notion that driver mutations give rise to neoplastic proliferation. The knowledge derived from whole-scale cancer genome sequencing has identified somatic mutational landscapes in cancer, while years of fundamental research in cancer genetics, signaling, and cellular and molecular biology have depicted various cancer-specific mutations, i.e., among others, *HER2* (breast), *EGFR* (colorectal, lung), *BRAF* (melanoma), *ALK* (lung), and *BCR-ABL* (leukemia), all used as therapeutic targets (reviewed in [5] and references herein). The BCR-ABL fusion protein has proved one of the most successful therapeutic targets thus far, promoting the design of imatinib (Gleevec) to treat chronic myeloid leukemia.

Decades later, the overall cancer mortality rate has declined since 1971 for most, though not all, cancer types, essentially due to improvements in prevention, surgical and radiation-based treatments, early detection, and a reduction in smoking [6]. In contrast, the chemotherapy war is still ongoing in an endless fight—one battle after another—testing myriads of potential therapeutic target genes which in principle should wipe out cancer cells. The drugs have, however, often shown opposite effects, eliminating chemosensitive cells and promoting the survival of chemoresistant ones, capable of adapting to novel environmental conditions and propagating with enhanced invasion potential. In that light, the war on cancer is not won and the anticipated victory seems to remain out of reach in spite of the deployed efforts [3].

Despite the accumulation over the years of an impressive amount of data from cancer types of different origins, the search for a common origin of cancer—another major scientific objective of the war—turned out to be a no-victory battle, leaving that question unanswered together with the legacy of a dramatic uncertainty about the directions to pursue (reviewed by [7]). The battle undertaken in 2007 with The Cancer Genome Atlas program (TGCA) (available at https://www.cancer.gov/about-nci/organization/ccg/research/structural-genomics/tcga) aimed for a systematic sequencing of the genomes of primary cancers; a major goal of TCGA was to generate an extensive atlas of cancer genomic profiles, with the expectation that such a comprehensive analysis would eventually reveal the genetic and mutational bases of cancer as a fundamental platform to design effective treatments. The sequencing data have, however, failed to identify universally shared driver mutations, and have revealed instead highly heterogeneous landscapes that challenge the somatic gene mutation theory of cancer [7,8,9]. As reported by Paul Nurse, the disappointment in such negative results was radically synthesized by Sydney Brenner with the sentence: “We are drowning in a sea of data and starving for knowledge” [10].

Those studies yielded further unexpected indications: (i) Cancer-associated driver mutations also occur in normal tissues, a large proportion of which involve “cancer genes”. Such mutations display signs of positive selection, suggesting that clonal evolution is in fact a ubiquitous phenomenon not limited to neoplasia, as traditionally thought (reviewed by [11]). (ii) Mutations in leukemia genes have been reported in the hematopoietic system of healthy individuals [12,13], and cancer-associated mutations have been found in a variety of tissues from healthy individuals, including skin [14], esophagus [15,16], colon [17], brain [18], and a set of 29 human tissues [19]. (iii) Almost every coding gene can be a “cancer gene” potentially associated with cancer in a particular context. A PubMed analysis showed that the majority of human genes (87.7%) have been studied in correlation with cancer, and the available literature supports their potential relevance to cancer [20]. That study re-evaluates the TGCA’s mission and raises the question of whether the concept of “cancer genes” is still acceptable in the current form. (iv) Noncoding RNAs (ncRNAs), including micro RNAs (miRNAs) and long noncoding RNAs (lncRNAs), have been implicated in chromosome instability in cancer. miRNAs have been found to target checkpoint proteins involved in modulating chromosome instability, while specific lncRNAs have been shown to act in chromosome instability-associated pathways [21]. (v) Conditions of genetic instability, favoring gross genomic alterations (e.g., aneuploidy and chromosomal alterations, with the possible outcome of chromothripsis), are overwhelming in many cancers and have a higher predictive prognostic power compared to genetic mutations [7,22,23].

Importantly, the large-scale cancer projects through which the human tumor genomes have been accurately scanned have identified heterogeneous, variable combinations and paths of mutations that failed to drive metastatic progression, although cancer clones expressing metastasis-associated traits were identified (extensively reviewed by [24,25]). These apparently contradictory results therefore call other players into question, suggesting that epigenetically controlled programs have roles in metastatic progression in the absence of driver genetic mutations. In support of this view is the finding that metastatic cells can be reprogrammed and that metastatic traits are reversible in embryonic microenvironments, implying that they are not caused by genomic mutations [26]. The reversibility of metastatic phenotypes implies strong links with epigenetic variations that do not affect the primary DNA sequence, but involve reversible phenomena, such as post-translational-dependent signaling, as well as DNA and chromatin modifications.

Consistent with this, the altered expression or dysfunction of chromatin modifiers and remodelers are reported in an ample variety of cancers, suggesting crucial roles for epigenetic aberrations in cancer (reviewed by [27]). In the frame of global projects, enormous advances have been made that have radically transformed the view of the genome. The Encyclopedia of DNA Elements (ENCODE) first showed that a small DNA fraction is transcribed into protein-coding mRNAs, while most of the genome is transcribed into non-coding RNA molecules, some of which have emerged as key regulators of gene expression [28]. Moreover, the International Human Epigenome Consortium (IHEC) is a scientific organization that coordinates the production of epigenome maps through the characterization of the regulatory, methylation, and transcription landscapes from a range of tissues and cell types to gain insight into the epigenetic control of cell states relevant for human health and disease [29]. We shall return to this in the next chapter. The technological and scientific advances underlying the ENCODE encyclopedia compilation have identified epigenetic regulators of gene expression in cancer via the in-depth characterization of genome domains, transcription factor associations, chromatin structure, and histone modifications [27,30]. Those findings in turn have rationalized the notion that cancer hallmarks [2,4] can originate from epigenetic dysregulation [31] in the absence of genetic alterations. Epigenetic mechanisms have also emerged as important players in the transition from primary to metastatic cancer cells, while mutations do not appear to provide discrete causal contributions to the process, as assessed by extensive sequencing efforts [32]. In summary, whole genome sequencing studies do not generally confirm the linear model of tumorigenesis through incremental mutational events, nor do they support the conclusion that metastatic cancer progresses from an initial pre-cancer lesion via subsequent additive mutations.

Overall, these data suggest that epigenome dysregulation may be at the heart of both cancer genesis and metastatic progression, even in the absence of genetic contributions. The emergence of the notion of the epigenetic origin of cancer paved the way to the concept of epigenetic therapy and to the development of novel “epi-drugs” to antagonize cancer progression via cytostatic/differentiating approaches rather than conventional cytotoxicity [33]. At present, epigenetic therapies are being successfully applied in the treatment of acute promyelocytic leukemia [34], while still in the preliminary, albeit encouraging, steps in treating solid carcinoma [35].

The definition of epigenetic landscapes in cancer biology calls for a radical revision of established theories and an undermining of the role of mutations in the genesis and progression of cancer [36].

## 2. The Epigenetic Impact of Reverse Transcriptase (RT) Inhibitors on Cancer

**Figure 1:**
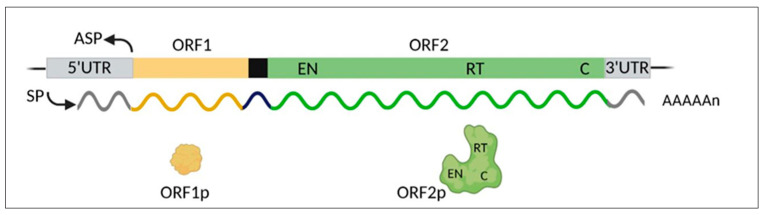
Schematic map of the human *LINE-1* full-length retroelement and encoded products. The main genetic regions include: *SP*, sense promoter; *ASP*, anti-sense promoter; *5′-UTR* and *3′-UTR*, untranslated regions; *ORF1* and *ORF2*, open reading frames 1 and 2. The black box represents the intergenic spacer between the two *ORF*s. The polycistronic primary transcript, and the encoded protein products, are represented in the same color code. The ORF2 product encompasses regions encoding endonuclease (EN), reverse transcriptase (RT), and cysteine-rich (C) domains. The image was created using BioRender (biorender.com).

A striking finding from the human genome sequence completion showed that coding genes account for a mere 1.2% of the genome, while the remaining portion is made of non-coding DNA [37,38]. That discovery marked a paradigm shift that has revolutionized the traditional concept of genome function and has disclosed an unexpectedly complex genomic landscape. In the wake of these findings, the non-coding DNA, long dismissed as “junk” [39,40], has turned out to regulate myriads of genome-wide functions. About 45% of non-coding DNA is made up of various families of retrotransposable elements (REs), or retransposons: the most abundant *LINE-1* family represents 17–20% of the human genome; *Alu* elements, 13%; human endogenous retroviruses (*HERVs*), 8%; and *SINE-VNTR-Alu* (*SVA*), 0.2%. *LINE-1*s are autonomously REs which amplify and propagate via a “copy and paste” mechanism mediated by reverse transcription, endonuclease, and RNA-binding activities, encoded by the open reading frames 1 and 2 (*ORF1* and *ORF2*) of full-length *LINE-1* ([41]; see Figure 1). In contrast, *Alu*s and *SVA*s lack these activities and use those provided by *LINE-1* for their non-autonomous mobilization. DNA transposons, mobile elements exploiting a direct “cut and paste” process, are less abundant, accounting for 3% of the human genome [37,41].

Human *LINE-1*s (Figure 1) comprise about 500,000 elements, most of which are truncated and unable to retrotranspose; it is estimated that only a sub-population of 80–100 *LINE-1*s are full-length and retrotransposition-competent, six of which are highly active (hot *LINE-1*s), accounting for 84% of the overall retrotransposition activity [42]. The RT enzyme is key to the *LINE-1* life cycle and retrotransposition. It is encoded by the *LINE-1 ORF2* region, within the ORF2p protein product, which also harbors an endonuclease (EN) domain capable of cleaving the DNA. *LINE-1* ORF1 protein (ORF1p) has RNA-binding activity of less well-defined function [41].

Both ORF1p and ORF2p proteins contribute to the *LINE-1* retrotransposition machinery, which has a high cis-preference for retrotranscribing its own *LINE-1* mRNA [43,44,45]. Other non-autonomous repetitive elements, e.g., Alu and SVA, also propagate via a reverse transcription step and rely on *LINE-1*-encoded ORF1p and ORF2p. The system is also used for the reverse transcription/retrotransposition of mRNAs that generate a large population of processed pseudogenes [46]. The advent of high-throughput technologies has shown that genomes are in fact crowded with sequences of reverse-transcribed origin, often correlated with the emergence of various pathologies that cannot be discussed here for reasons of space, but have been extensively reviewed regarding their circumstances and underlying mechanisms [47,48,49,50].

A correlation between RT activity and cancer was first theorized by Temin in 1971 [51] and experimentally confirmed a few years later [52]. That notion is now supported by a large body of evidence: many human cancers are characterized by *LINE-1* de-repression [53], and massive somatic *LINE-1* retrotransposition ([54]; reviewed by [48,55]). It has long been known, and is now well established, that cancer cells are characterized by aberrant genome-wide DNA hypomethylation, which entails the de-repression of retrotransposons [56]; reviewed by [57]. The hypomethylation of *LINE-1* favors their increased transcription, product translation, and hence mRNA retrotranscription, leading to amplification and insertional retrotransposition, a situation that is associated with a poor prognosis in various cancer types [58]. While these processes are well documented and are extensively reviewed elsewhere ([57] and references herein), the specific mechanisms through which the activation of *LINE-1*s acts in cancer is still being debated [49], and it remains to be established whether insertional mutagenesis is a cause or a consequence of tumorigenesis.

A link between *LINE-1* activity and cancer has also emerged from studies of the transgenic mouse strain MMTV-PyVT (Mouse Mammary Tumor Virus–Polyoma Virus T antigen), a well-defined model of breast cancer progression [59]; in that model, the RT activity was found to increase linearly in breast cancer compared to normal tissues, *LINE-1* expression was significantly upregulated—although not linearly—and *LINE-1* copy number also increased until reaching a plateau [60]. These events occurred early during cancer onset, before the appearance of recognizable histological alterations and tumor marker expression: this suggests that the activation of *LINE-1* machinery is not a passive consequence of tumor growth but, rather, acts early in the cancer-promoting process.

It is worth remembering that *LINE-1*s are stress-responsive elements [61,62]. Their activation can therefore be triggered by both cancer-inducing stressing conditions, i.e., under the same stimuli that initiate cancer, and by the loss of control on RE activity typical of cancer cells [63,64]. The RT enzymatic activity plays an active role in the process. Indeed, evidence from our [65,66,67] and other groups [68,69,70,71,72,73,74] indicates that nonnucleoside RT inhibitors (NNRTIs), including nevirapine and efavirenz (EFV), used in AIDS treatment as inhibitors of the HIV-derived RT, possess anticancer efficacy in several systems: they reduce proliferation and induce apoptosis in cancer-derived cell lines (melanoma, glioblastoma, osteosarcoma, prostate, colon, and thyroid cancer), often accompanied by the restored expression of lineage-specific differentiation markers, while exerting mild or no effects on non-cancer cells; furthermore, they antagonize cancer progression in animal models. EFV has also been successfully tested in a human phase-II trial with metastatic prostate carcinoma patients [75,76], paving the way to its possible use in a non-cytotoxic cancer treatment. In retrospect, these data are consistent with epidemiological evidence that AIDS patients treated with highly active antiretroviral therapy show a reduced incidence of AIDS-related cancers (e.g., Kaposi sarcoma, lymphoma, etc.) [77,78,79,80]. Recently, a newly designed NNRTI compound specifically targeting the human RT, called SPV122 [81,82], proved more effective than EFV in inhibiting cancer growth, both in cell lines and in animal models [83]. Importantly, the anticancer effects exerted by all tested NNRTIs are reversible: cancer progression is quickly resumed on discontinuing the treatment and returning the cells back to a drug-free medium [67,83,84]. Clearly, the reversibility of cancer inhibition would not be compatible with a retrotransposition-based effect generating *LINE-1* new retrotransposal insertions. Rather, it would appear that cancer onset and progression are under an epigenetic control system of which *LINE-1*s are a crucial component. Anticancer effects have also been reported for nucleoside RT inhibitors (NRTIs) [85,86]. Consistent with these observations, The induction of downregulation of *LINE-1* expression by RNA interference in cancer cells also yielded a decrease in proliferation rate, a differentiated cell morphology, and the restored expression of marker genes, as well as a decrease in cancer cell tumorigenicity in mouse models [67,84]. All these effects parallel those observed with RT inhibitors. Significantly, A-375 melanoma cells with stably interfered *LINE-1* expression showed a lower tumorigenic potential compared with non-interfered cancer cells when inoculated in nude mice [84]. In contrast, no anticancer activity was observed when downregulating the expression of *HERV-K* endogenous retroviruses. These findings therefore support the conclusion that the retrotranscription of *LINE-1*, but not of endogenous retroviruses, plays a causative role in the genesis of cancer, and pinpoint an epigenetic level of control distinct from the genomic mutations induced by retrotransposon insertions.

Consistent with this, EFV was found to induce a global reprogramming of the transcription profiles in cancer cells, including protein-coding mRNAs, as well as micro RNAs (miRNAs) and ultra-conserved region (UCR) long non-coding RNAs [87]. Specifically, EFV reversed the expression of a miRNA class called metastamiRs—a subpopulation of miRNAs with crucial roles in tumor progression, invasiveness, and metastasis—by upregulating species that are underexpressed in cancer cells and, conversely, downregulating overexpressed ones. The data suggest that this transcriptional reprogramming is mediated by RT-dependent RNA:DNA hybrids which, importantly, are abundant in cancer cells, where RT expression is high, and undetectable in healthy cells, which express low or no RT activity. We interpret these data as indicating that, in cancer cells, the RT encoded by highly active *LINE-1*s reverse-transcribes the available RNA populations, yielding abundant RNA:cDNA hybrids to the detriment of double-stranded RNA, which normally forms in healthy cells, either via intramolecular base paring of an RNA containing two oppositely orientated retroelements, or via base pairing between sense RNA and antisense RNAs to be processed by Dicer into miRNAs. Treatment with RT inhibitors abrogates the formation of RNA:DNA hybrids in cancer cells and restores the “healthy” expression profile, accompanied by the epigenetic conversion to a normal phenotype [87]. These results hint at a functional connection between miRNAs and *LINE-1*s, in agreement with the notion that *LINE-1* actually originates a proportion of cellular miRNAs [88,89]. Together, these data provide new clues towards an understanding of the *LINE-1*-based cancer-promoting mechanism.

## 3. RT Inhibitors Induce Epigenetic Alterations in the Nuclear Architecture of Cancer Cells

The finding that the NNRTIs EFV and SPV122 induce the reprogramming of the transcription profile in cancer cells raises the question of whether the nuclear landscape is reshaped in parallel. In agreement with that prediction, we recently found that RT inhibitors induce extensive changes in the nuclear architecture of PC3 metastatic prostate carcinoma cells, i.e., the increased methylation of peripheral heterochromatin at histone H3 (H3K9me2), DNA damage with induction of Chk2 and p21, disruption of lamin B1, and formation of DNA-containing micronuclei [90]. These effects are reversible, as cells reconstitute the integrity of lamin B1 and re-establish their original cancer cell features on discontinuation of the NNRTI treatment. The lamina, a protein meshwork lining the inner nuclear membrane, is largely—though not exclusively—associated with heterochromatin and genomic regions to which it confers an overall repressive environment [91,92]. These regions, forming the lamina-associated domains (LADs), are enriched in *LINE-1* elements [93,94] and contribute to the spatial organization of the genome and control of nuclear transcription [91,95]. The implications of these findings in the genesis of cancer will be discussed in depth in the next section. Remarkably, a recent study in the context of progeroid syndromes, which are basically caused by lamina defects, has depicted a novel role of *LINE-1* RNA transcription in influencing chromatin organization, cell proliferation, DNA damage, and gene expression profiles [96].

In evaluating the effects of RT inhibitors in inducing nuclear damage and reorganization, it is worth noting that RTIs act not only as inhibitors of *LINE-1*-encoded RT activity, but—somewhat unexpectedly—they upregulate transcription of *LINE-1* mRNA (our unpublished data), acting therefore as stress-inducing agents. Antiretroviral drugs are reported to impact autophagy [97]. Consistently, we have found that NNRTIs trigger an autophagic response, reflected by increased marker expression (LC3, Beclin-1, ATG7, and p62), and sensitivity to the autophagy inhibitor 3-methyladenine [90]. Concomitantly, NNRTIs induce genomic DNA damage, another powerful inducer of the autophagic response [98,99]. Autophagy in turn degrades retrotransposon RNA [100], indicating a functional interplay between stress- or NNRTI-induced *LINE-1* upregulation and the autophagic response. Prolonged exposure to NNRTIs also induces apoptotic death in cancer cells. Autophagy and apoptosis are molecularly and functionally distinct processes, yet both can concur in the response to stressing stimuli [101]. The available data thus far suggest that autophagy contributes to the anticancer mechanism of RT inhibitors. Remarkably, in our experiments only cancer cells proved sensitive to the antiproliferative and lamin-disruptive effects of NNRTIs, while non-cancer cell lines (WI-38 fibroblasts, hTERT/RPE-1 epithelial cells, and PNT2 normal prostate epithelium) remained unaltered in proliferation capacity and lamina integrity. These differential responses correlate well with the different extent of *LINE-1* expression, generally upregulated in cancer cells and very low in healthy differentiated cells. Thus, the selective response of cancer cells, but not healthy cells, to NNRTIs likely reflects their higher level of *ORF2*-encoded RT activity, while the latter express low levels of this target. The ability of NNRTIs to target cancer but not healthy cells hints at an important advantage in the perspective of potential therapeutic applications. Overall, the data highlight a multifactorial chromatin-shaping *LINE-1*-dependent mechanism active in cancer cells.

## 4. The Epigenetic Reshaping of the Cancer Genome: A Model

**Figure 2:**
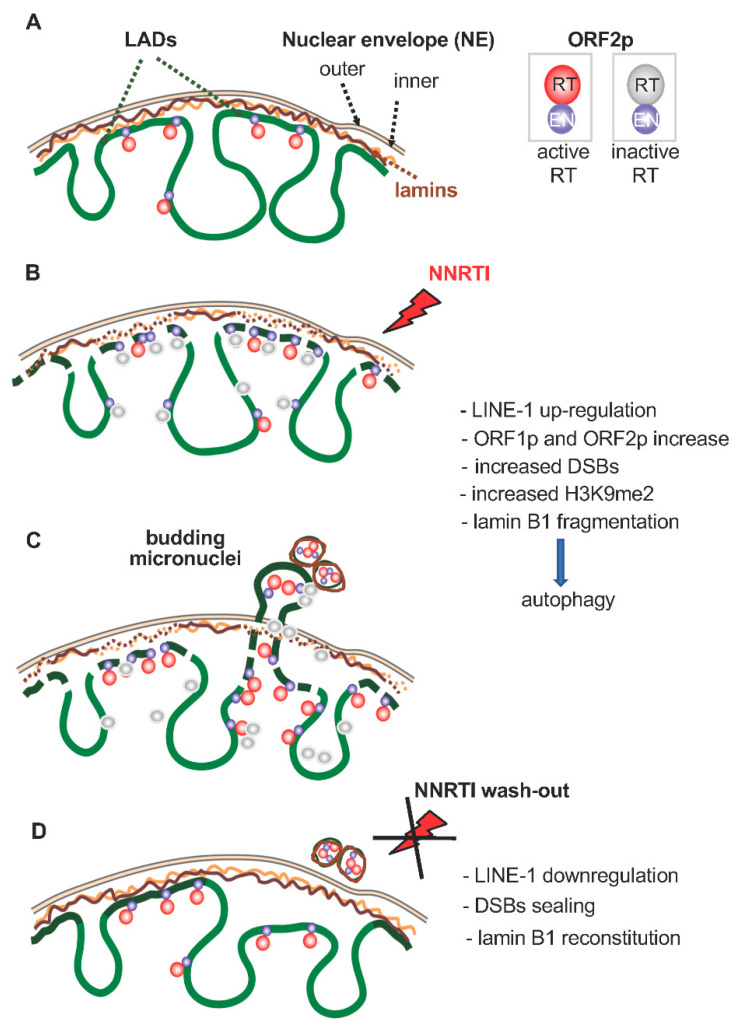
A hypothetical model for *LINE-1*-mediated chromatin remodeling. (**A**) A simplified representation of chromatin–lamin interactions. LADs: lamin-associated domains of chromatin. Chromatin is schematized in green. For simplicity, the nuclear envelope (NE) is represented without nuclear pores. (**B**) NNRTIs act as stressing stimuli (red flash): in response, *LINE-1* products ORF1p and ORF2p are upregulated, the DNA is cleaved (DSBs, double strand breaks), lamin B1 is fragmented, and autophagy is activated. (**C**) DSBs and lamin B1 fragmentation increase chromatin plasticity and remodeling. Concomitantly, DNA- and lamin B1-containing micronuclei form, are encircled by the NE, and released outside nuclei. (**D**) After the removal of the NNRTI stress, the nuclear structure is stabilized in a remodeled conformation, driving the formation of novel genomic circuits.

The cascade of events induced by stressing conditions constitutes the core of the proposed mechanism for the role of *LINE-1* in cancer. Figure 2 schematically illustrates a hypothetical model for the epigenetic reshaping of the genome induced by stressing stimuli. In our work, NNRTIs were used as stressing tools, but the model can apply to myriads of other stressing sources. In the first step, chromatin organization is rearranged in cancer cells, particularly at LADs, represented in Figure 2A, in response to stressing stimuli. The nuclear envelope provides the platform where these changes are dictated. Stressors act at multiple levels: they promote upregulation of *LINE-1* expression [58,59], concomitant with the formation of unrepaired DNA breaks, possibly reflecting the upregulation of *LINE-1* ORF2p-encoded EN activity. Under normal conditions, EN-generated DNA cleavage is “repaired” by the insertion of retrotranscribed DNA. In the presence of NNRTIs, that activity is inhibited and becomes uncoupled from the increase in EN. Associated with this are an increased heterochromatinization at the nuclear periphery, nuclear lamina fragmentation, and activation of the autophagic response (Figure 2B). The combination of these events generates conditions that increase nuclear plasticity and facilitate genetic instability, as evidenced by the formation of micronuclei released from the cancer cells [90] (Figure 2C). At this stage, the nuclear architecture is in a “variation-prone” condition, with the potential to remodel the epigenetic landscape, and highly permissive towards the incorporation of stress-induced changes. When cells are no longer under stress, or—as in our experimental context—NNRTIs are washed out (Figure 2D), the lamina integrity is restored and the reshaping is complete. Structural novelties that may have been incorporated are stabilized in the nuclear reorganization, which now diverges from the initial condition (as in Figure 2A). The model predicts that, when exposed to appropriate stressing conditions: (i) a normal cell can be transformed into a cancer cell, (ii) a cancer cell into a metastatic cell, and (iii) chemotherapy-resistant cancer cells can be generated through a pure epigenetic process not requiring genomic mutations. The reshaping of the epigenetic landscape impacts on transcription profiles and promotes the formation of novel genomic circuits coding for cancer phenotypic traits. In evolutionary terms, this is an epigenetic adaptive response to stressing stimuli, able to induce substantial phenotypic variations and to re-orient the fate of the cells.

## 5. Bursts of *LINE-1* Expression in the Early Genesis of Metastasis

With the remarkable exception of lineages of the central nervous system, in which *LINE-1*s are actively expressed (reviewed in [102]), *LINE-1* transcription is absent, or very low, in normal differentiated cells, while being upregulated in cancer, depending on endogenous and exogenous stressors. Based on the data discussed above, we propose that the differential activation of stress-responsive *LINE-1* expression can generate the heterogeneity among differentiated cell populations typical of human cancers [103,104]. It may be speculated that a single, genome-wide burst of *LINE-1* expression promotes the emergence of heterogeneous cell populations with varying degrees of malignancy, a proportion of which likely contribute the so-called “primary tumor cells”, while other—less abundant—cell populations would be primed to generate more aggressive cancer cells or, in due time, metastatic cell populations [105]. In light of the mechanism sketched in Figure 2, different levels of *LINE-1* expression would generate different levels of nuclear architecture disruption and correspondingly different degrees of malignancy. Accordingly, a metastatic cell would not necessarily represent the final product of an evolving cancer cell via the additive accumulation of driver mutations over time, but would be primed already at cancer onset by sub-populations of cells endowed with traits determining high degrees of malignancy. The model is inspired by the “Big Bang” hypothesis of colorectal cancer, in which a single clonal expansion originates the heterogeneous population of cancer cells, some of which appear “born-to-be-bad”, expressing clear traits of early malignant potential which would then progress and expand in parallel [106]. A burst of *LINE-1* expression may well represent that ancestral triggering event.

Along the same lines, the emergence of cancer subpopulations with acquired chemoresistance could be similarly explained. Chemotherapeutics are stress-inducing agents and, as such, can be sensed by the genome-wide *LINE-1* system. *LINE-1* can be upregulated in response and trigger the described cascade of events that increase chromatin plasticity. Chromatin reshaping [107], together with the remodulation of coding [108] and non-coding genes [109], are consequential events in drug-treated cells, causing profound changes in their behavior and fate. Cancer cells are adaptive systems and it has been suggested that the acquisition of drug resistance is an adaptive response, mediated by a Lamarckian-type of induction rather than by a Darwinian-like selection of genetic mutations [110]. In other words, the resistant malignant cells would not represent the selected population by the chemotherapeutic treatment, but, rather, evidence suggests that the treatment induces cells to become resistant via epigenetic mechanisms [111,112,113]. This view is supported by evidence showing that refractoriness to drug treatment, at least in some cases, can be reversed by epigenetic reprogramming: thus, non-genetic induction may be functionally predominant over genetic alterations ([113] and references herein). The cancer genome-reshaping mechanism proposed in this model might be the source of epigenetic variations and, possibly, contribute to establish cancer resistance.

We would like to note that the model proposed here, by and large, is consistent with experimental evidence published in two very recent studies of a large number of colorectal cancers [114,115]. We expect that the novel view emerging from these studies might indeed apply to cancers of many different origins.

## 6. The Entangled Worlds of Embryos and Cancer: Cancers Stem from the Reactivation of the Embryonic Epigenetic Circuits

A peculiar aspect of cancers is that, although they might be seen as aberrant overgrowing cellular jumbles, their progression occurs in fact through trajectories with recurring features. The conceptualization of cancer hallmarks mentioned in the introduction to this review [1,2,3,4] sees phenotypic plasticity and the disruption of the cell differentiation program as key players in cancer. Indeed, regardless of the many possible causes that may have triggered cancer onset, and the variety of mechanisms operating in targeted tissues of different histological origin, some of the shared hallmarks of cancer cells, i.e., loss of differentiation, unrestrained cell growth, invasion, genomic instability, DNA hypomethylation, reprogrammed gene expression, increased expression of retroelements (*LINE-1*s and *HERV*s), closely resemble those characterizing early embryos (reviewed in [116] and references herein). That analogy recalls Virchow’s observation of the mid-19th century, first noting that tumors share common features with embryos [117]. The embryo/cancer analogy concerns fundamental functions, e.g., cell growth, differentiation, and gene expression, suggesting a common underlying mechanism in both contexts. In support of this view, well-established examples of genes active during embryonic life, and silenced upon differentiation in adults, are reactivated in tumorigenesis [118,119,120,121]. To mention one example among others, the morphogen Nodal of the TGF beta family, which is crucial in organizing the early embryonic axis [122], is re-expressed at high levels in several cancer types, including melanoma, breast, and pancreatic cancers, where it acts as a driver of tumor growth and cellular plasticity [123]. There is a direct correlation between Nodal expression levels and the tumor grade, consistent with growing evidence that Nodal can be an effective therapeutic target of “epi-drug” treatment [124]. Moreover, among the many genes essential for embryo and cancer cell growth, *BRD4, CDK9, HDAC3*, and *mTOR* encode protein products for which specific inhibitors have been designed and enrolled in clinical trials as therapeutic reagents [124].

Generalizing this notion, we speculate that the unscheduled reactivation of genetic circuits, active throughout embryogenesis and subsequently silenced in adult tissues, is an epigenetic trigger of cancer onset. The reactivation of embryonic genetic circuits underlying the developmental program delivers “embryonic” information in the incompatible context of differentiated cells: the conflicting collision between embryo-specific expression and the “wrong” context of differentiated cells drives cells to develop a cancer phenotype, which may be seen as a failed attempt of differentiated cells to revert to an embryonic state. Relevant to this point is the recent spatially resolved multi-omics analysis of a collection of colorectal cancer samples, showing the resumption of embryonic genes, described by the authors as follows: “One of the most intriguing results was the evidence of reactivation of developmental genes during tumorigenesis” [115].

**Figure 3:**
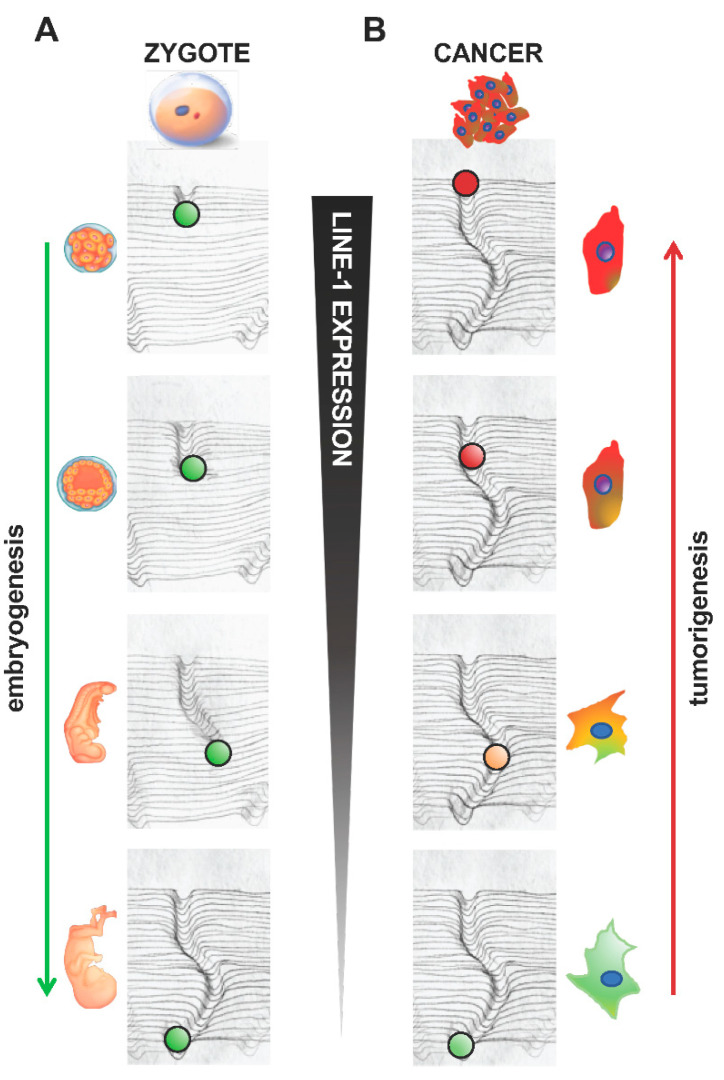
Reactivation of embryonic genetic circuits in differentiated cells triggers cancer onset: a model. Schematic illustration, inspired by Waddington’s model, representing the following: (**A**) The process of the canalization of the epigenetic landscape during embryogenesis; the canal formed by the green ball rolling from the top of the hill represents new genomic circuit(s) shaping the epigenetic landscape. (**B**) Aberrant reactivation of embryonic gene expression in differentiated cells (green cell, bottom right panel) triggers cell de-differentiation and the formation of heterogeneous cell populations with different degrees of malignancy (shades from yellow to red). The extent of malignancy would depend on how far back the cell retraced the canalized circuit, reflected by varying extents of de-differentiation. Correspondingly, *LINE-1* expression is high in early embryos and in de-differentiated cells, and very low in differentiated cells and tissues.

In a hypothetical model, the cancer-embryo interweaving illustrates the concept that the root of cancer consists in tracing back the differentiation process of the cells whose epigenetic landscape was modeled during embryogenesis. Figure 3 schematically represents this model, inspired by the “hills and valleys” of Waddington’s metaphor [125]. Variations in the epigenetic landscape occur during embryonic development through the formation of “canalized” genomic circuits, triggered soon after fertilization, and further finely tuned throughout embryogenesis. In Figure 3A, novel trajectories are traced—represented by the ball rolling down from the top of the hill and forming the newly “canalized” genomic circuit—while development unfolds. At the bottom of the hill, the cell fate is completed, development is achieved, and new individuals are formed. At this stage, the embryonic canalization has served its purpose and is functionally inactivated. However, it is not permanently erased but is stored in the epigenetic memory of the cells. In differentiated tissues, ”adult” canalized circuits replace the “embryonic” ones. Figure 3B illustrates cancer cell formation as the product of de-differentiation, during which adult somatic differentiated cells retrace, in the opposite direction, the paths walked during embryogenesis. Such reactivation of the embryonic canalization restores embryonic features in the cells by remodeling the epigenetic landscape, accompanied by the reprogramming of gene expression and the induction of genome instability. Unscheduled bursts of *LINE-1* expression, and the ensuing activation of chromatin-remodeling mechanisms (see previous section), may induce the reactivation of embryonic circuits and promote cancer progression toward higher degrees of malignancy. This epigenetic process does not necessarily require the contribution of gene mutations. The transition from adult to embryonic features would thus be an “easy” shift, only relying on the resumption of pre-existing circuits in the epigenetic memory of the cells and poised to be reactivated.

In conclusion, the evidence summarized in this review sees the implication of *LINE-1*s in cancer via mechanisms that are independent from, and complementary to, their insertional mutagenesis activity. These mechanisms rely on the capacity of *LINE-1* elements and protein products to act at the epigenetic level in reformatting the genome organization and expression along pathways that were once traveled during embryogenesis, the “resurrection” of which reprograms the expression profiles of adult cells. The knowledge emerging from these studies might pave the way to novel clinical opportunities based on RTIs as novel therapeutic tools. RTIs might in certain cases replace conventional cytotoxic chemotherapy, or be used in cooperation with it, ideally potentiating its effects, and enabling lower doses to be effective, thus decreasing secondary effects and unspecific toxicity, which is one of the compelling objectives of the war on cancer. As pointed out by Hanahan [3], therapeutic strategies should avoid multiple diversifications targeting numerous substrates each of which would identify a specific cancer. Rather, the therapeutic “magic bullet” should hit few targets shared by a large number of cancers. RTIs would appear to fulfill this idea, leading to the elimination of ORF2p-overexpressing cancer cells while sparing all others.
